# Development and validation of the cancer self-perceived discrimination scale for Chinese cancer patients

**DOI:** 10.1186/s12955-018-0984-x

**Published:** 2018-08-14

**Authors:** Lin-sen Feng, Xin-yue Li, Hong-rong Wang, Jing-jing Zhan, Dong Chen, Yu-feng Wang

**Affiliations:** 1 The Sixth Affiliated Hospital of Kunming Medical University (The People’s Hospital of Yuxi), Yuxi, 653100 Yunnan China; 2 The Third Affiliated Hospital of Kunming Medical University (Yunnan Cancer Hospital), Kunming, 650118 Yunnan China; 3 The People’s Hospital of Jiangchuan District, Yuxi, 652600 Yunnan China

**Keywords:** Cancer self-perceived discrimination scale, Psychological problems, Self-perceived discrimination

## Abstract

**Background:**

To develop a Cancer Self-Perceived Discrimination Scale (CSPDS) for Chinese cancer patients and to assess its reliability and validity.

**Method:**

A total of 178 patients were recruited and the classical test theory was used to develop the CSPDS. Item analysis was adapted to improve the preliminary version of the CSPDS, then the reliability, the validity and the acceptability of the final version of CSPDS were assessed.

**Results:**

This CSPDS contained 14 items classified into 3 subscales: social withdrawal with 7 items, stigma with 4 and self-deprecation with 3. Good validity (χ^2^/df = 1.216, GFI = 0.935, AGFI = 0.903, I-CVIs> 0.80) and good reliability (Cronbach’s alpha = 0.829, Spearman-Brown coefficient = 0.827, test-retest reliability coefficient = 0.944) were found. The completion time was 6.06 ± 1.80 min. Participants who were female and reported poor self-rated health tended to have higher CSPDS scores (*P* <  0.05).

**Conclusions:**

The results indicated that this CSPDS could be used to assess the level of self-perceived discrimination and to preliminarily screen perceived discrimination among Chinese cancer patients, especially in Southwest China. It may provide a basis for scientific assessment of targeted patient education, psychological counseling, social interventions, and psychotherapy in the future.

## Background

Self-perceived discrimination, which is also called “perceived discrimination”, is a type of negative psychological status. Once a group member begins to experience a process in which one is treated differently (especially unequally or disparagingly) [1], self-perceived discrimination, originating from the person’s subjective feelings, is likely to arise (either intermittently or continually). It can reflect the level of actual discrimination or systematic over- or under-estimation of actual discrimination [2].

Discrimination exists in a variety of forms, for example, gender, race, income, social class, workplace, insurance, disease, etc. [3]. Disease-related discrimination refers to how healthy people intentionally exclude individuals with certain diseases or disabilities, thereby leading to isolation, prejudice, and discrimination towards them. Most literatures on disease-related discrimination have focused on pathogen carriers and patients with specific chronic infectious diseases (e.g., tuberculosis, AIDS, leprosy) [4–7], or on patients with mental disorders such as depressive disorder or schizophrenia [8, 9]. Disease-related discrimination can come from both general population and medical professionals, so it is widespread and worthy of attention [10].

Cancer has become a common chronic disease worldwide [11, 12]. The prevalence of long-term psychological effects of cancer cannot be neglected [13, 14], which attaches more importance to cancer-related self-perceived discrimination [15]. Patients can perceive discrimination due to fear of death and side effects from cancer treatments [16–19].

Previous literature showed that cancer discrimination might originate from workplace and insurance. Workplace discrimination mainly included hiring discrimination, refusal of workplace accommodations, limited career advancement opportunities, and so on [20]. Genetic discrimination was another issue cancer patients are facing [21]. Researches had revealed that there were relations between cancers and gene mutations. For instance, hereditary breast and ovarian cancer (HBOC) was associated with mutations in BRCA [22]. In these patients, fears of health insurance discrimination and life insurance discrimination were well documented, which was the proof of the existance of genetic discrimination in insurance [21].

However, there is no proper measure to assess cancer self-perceived discrimination since there has been only a very few scales specifically evaluating stigma and shame. Cataldo JK designed the Cataldo Lung Cancer Stigma Scale (CLCSS) with 31 items and four subscales (stigma and shame, social isolation, discrimination and smoking) based on the HIV Stigma Scale, and he paid more attention to the relationship between smoking status and lung cancer stigma [23]. Kissane DW developed a 20-item Shame and Stigma Scale (SSS) for head and neck cancer which contained 4 subscales: shame with appearance, sense of stigma, regret, and speech/social concerns, and he focused on shame and stigma after alteration of body image and functions [24]. These scales had excellent reliability and validity, but they only focused on stigma which was just a part of cancer-related discrimination and were limited to a single type of cancer.

The purpose of our study was to develop a comprehensive Cancer Self-Perceived Discrimination Scale (CSPDS) to assess the level of self-perceived discrimination among Chinese cancer patients.

## Methods

### Study design and participants

The classical test theory was adapted, and the steps were as follows:

1. Develop a preliminary CSPDS based on semi-structured interviews, literature reviews, expert consultations and cognitive interviewing.
2. Collect data of the hospitalized cancer patients interviewed using the preliminary CSPDS.
3. Generate final version of CSPDS using item analysis.
4. Validate the final CSPDS.
5. Subgroup-analysis of the final CSPDS.

The inclusion criteria were as follows: patients were/had

- ≥ 18 years old;
- with cancers confirmed by pathology;
- aware of their own diagnosis for at least one month;
- volunteered for participation;
- the ability to complete the survey on their own or with the help of investigators;
- basic communication skills and understanding capability.

The exclusion criteria were as follows: if patients

- had a past or present chronic infectious disease or mental disorder;
- had severe vision or hearing loss, cognitive dysfunction, or disturbance of consciousness;
- had been critically ill and undergone emergency medical treatment.

Another additional inclusion criterion, having experienced discrimination after diagnosis for more than one month, was included during the semi-structured and the cognitive interviews.

This was a single-center study. All participants were from the Third Affiliated Hospital of Kunming Medical University (Yunnan Cancer Hospital), Kunming, Yunnan Province, Southwest China.

### Quesitonaire development and item selection

#### Step 1

The “information-saturated method” was adopted to determine the sample size, therefore, the patient samples depended on whether there was any further information in the semi-structured interviews [25]. The semi-structured interviews were concluded once no more information emerged. Eleven cancer patients participated in the semi-structured interviews (August 1 – September 10, 2014), among which 5 were from urban areas and 6 from rural areas; 4 out of these 11 accepted higher education, 5 secondary education and 2 primary education; 4 out of these 11 came from families with economic poverty and the rest are well-off. To ensure interviewees’ privacy and improve the authenticity of the data, the interviews were conducted face-to-face by professionals in a one-on-one capacity. Each interview was finished within 45 min. As most interviewees refused sound recording, all interviews were recorded by transcripts. The interviews focused on self-perceived discrimination after cancer diagnosis.

Standard definitions of discrimination and cancer-related discrimination were provided to the interviewees. The definition of discrimination is treating a person or a particular group of people differently, especially in a worse way compared with the way in which one treats other people, because of their skin colour, sex, sexuality, etc. By that analogy, the definition of cancer-related discrimination is treating the cancer patients differently, especially in a worse way compared with the way in which one treats other people, because of their cancer, disease manifestations, treatment-related toxicity, etc. Sample questions included “Have you ever experienced discrimination because of your disease?” “What kind of discrimination did you experience?” and “What was the impact of that discrimination?”. The interviewer had to make sure that interviewees did not wander off topic and interview contents were fully recorded. If at least three interviewees complained of the similar issues related to discrimination, we extracted them into the item pool. The interviews were analyzed and transcribed into text by two language experts in concise and understandable language as the final items.

#### Step 2

Literature on disease-related discrimination, particularly cancer, infectious disease, and mental disorders, was reviewed (August 5–September 3, 2014) [23, 24, 26, 27]. Language design of the item pool was borrowed from the literature reviewed, including grammar and the sentence patterns. Then the item pool was developed through the semi-structured interviews and literature reviews.

A five-point Likert scale was used for item responses. For the forward-scored items, the options were as follows: 1 = strongly disagree, 2 = somewhat disagree, 3 = neither agree nor disagree, 4 = somewhat agree, and 5 = strongly agree; for the reversed-scored items, the scoring was reverse. Thus, higher scores indicated stronger levels of self-perceived discrimination.

#### Step 3

Four oncologists and three experienced nurses were invited to assess the suitability of the items twice (September 19–September 30, 2014). After the first consultation, those inaccurate words or items were further adjusted and revised. The second consultation was to calculate the item-level content validity indexes (I-CVIs) [28]. The suitability of each item was reviewed and judged by the experts independently, with a 3-point rating scale (3 = completely relevant to 1 = completely irrelevant). If the index was < 0.8, we would repeat the former steps until it was ≥0.8.

#### Step 4

The cognitive interviewing was conducted to ensure whether all of the items were applicable for formal investigations. Twenty interviewees were invited for verbal probing, which was a method of cognitive interviewing with patients (October 10–October 25, 2014). In the first round, 10 interviewees finished every item in the scale and answered the question “can you repeat the items you just read in your own words?”. Then we revised relevant words based on the first round. Next, 10 other interviewees participated in the second round of verbal probing to ensure that each item IS concise, clear, accurate and unambiguous. If any ambiguous words existed, we would revise and invite another 10 patients to run the cognitive interviewing again.

### Data collection

The questionnaire is composed of three parts: an assessment of sociological characteristics (gender, age, marital status, residence, number of family members, average monthly family income, educational background, occupational classification, medical insurance, and self-rated health status), the preliminary CSPDS, and the basic clinical information (diagnosis and treatments). A common recommendation for the minimum necessary sample size for a factor analysis is *N* = 100, with a subject-to-variable ratio of no less than 5 [29, 30].

To create a quiet atmosphere and avoid interference, the questionnaire was administered during non-visiting hours. There were six investigators including oncologists, medical postgraduate students, and clinical medicine undergraduates, who were all qualified in unified training. Before issuing questionnaires, the inclusion criteria and exclusion criteria of formal investigations were used to identify the potentially eligible patients through hospital records. All participants were inpatients. In principle, the questionnaire was completed by participants themselves. When requested, investigators read the items to the patients but the patients provided the answers (usually performed when the patient had limited mobility). The formal investigation was done between November 2014 and February 2016.

### Analysis

#### Step 1: Item analysis

Pearson correlation coefficient was used to examine correlations between each item’s score and the total score, and items would be removed if the correlation coefficient was small (γ <  0.3) or was insignificant (*P* >  0 .05), or if it lowered the Cronbach’s alpha of the scale.

#### Step 2: Validity

The construct validity of the final CSPDS was verified using confirmatory factor analysis (CFA) and structural equation modeling (SEM). According to the developing design of the CSPDS, we proposed two models for validation: an uncorrelated three-factor model and a correlated three-factor model. The good model-fitting degree included probability level (*P* >  0.05), Chi-Square divided by degrees of freedom (χ^2^/df <  2), goodness of fit index (GFI > 0.90), adjusted goodness of fit index (AGFI > 0.90), comparative fit index (CFI > 0.95), normed fit Index (NFI > 0.90), Tucker-Lewis index (TLI > 0.95), and root mean squared error of approximation (RMSEA < 0.05) [31, 32]. The content validity was confirmed using I-CVIs and Pearson correlation analysis.

#### Step 3: Reliability

Cronbach’s alpha and Spearman-Brown coefficient with a minimum of 0.70 suggest a good internal reliability [33, 34]. Test-retest reliability was verified using intra-class correlation coefficients (ICCs), with an interval of one week between the first and the second test. An ICC greater than 0.80 indicates that the two tests had excellent test-retest reliability [35].

#### Step 4: Acceptability

The readability score was valued using Flesch Reading Ease and Flesch-Kincaid Grade Level (Office Word 2013, Microsoft Corp., USA), and the survey completion time was recorded.

#### Step 5: Subgroup-analysis

*The* CSPDS subgroup analysis based on gender, age, marital status, residence, family numbers, family income, education, occupation, self-rated health, time since diagnosis, medical insurance and cancer type was performed, using a one-way analysis of variance and Fisher’s least significant difference test (LSD-t) as well.

The statistical analysis was performed using Stata 12.0 (Stata Corp., USA) and Amos 19.0 (IBM, USA). The two tailed *P* ≤ .05 was considered significant.

## Results

### Sample characteristics

Two hundred ten cancer patients were approached, and 190 patients consented to participate, among which 178 patients completed the survey (the valid response rate was 93.68%). Among the 178 participants, 53 patients had lung cancer, 45 had gynecologic malignancies (including 24 ovarian cancer, 9 endometrial cancer, and 12 cervical cancer), 22 had breast cancer, 21 had head and neck cancer, 21 had gastrointestinal cancer, and 16 had lymphomas. The mean age was 53 years old (range: 19–80 years old). The mean time from the diagnosis to the interviews was 11.5 months (range: 1–204 months). Detailed information about the sample characteristics is shown in Table 1.

**Table 1:** Scores of participant characteristics (Mean ± SD)

Characteristics	*N*	Total scores	*P*
Gender			
Male	75	35.28 ± 11.07	0.007
Female	103	39.99 ± 11.51	
Age			
18–45	39	38.62 ± 9.90	0.128
46–59	88	39.32 ± 11.30	
≥ 60	51	35.27 ± 12.77	
Marital status			
Married	159	37.61 ± 11.08	0.186
Unmarried	19	41.32 ± 14.72	
Residence			
Urban area	116	38.10 ± 11.44	0.877
Rural area	62	37.82 ± 11.79	
Family numbers			
≤ 3	91	37.46 ± 11.14	0.521
≥ 4	87	38.57 ± 11.96	
Average monthly family income			
≤ ¥3000	78	39.04 ± 12.77	0.352
¥3001–6000	60	38.13 ± 10.30	
≥ ¥6001	40	35.80 ± 10.66	
Education			
Junior middle school and below	91	37.25 ± 13.08	0.371
Senior high school and above	87	38.79 ± 9.66	
Occupational classification			
Retiree	59	38.07 ± 11.98	0.066
Civil servant or institution officer	38	40.34 ± 6.89	
Farmer or migrant worker	53	38.91 ± 12.91	
Freelance professional	28	33.00 ± 11.91	
Self-rated health			
Good	41	33.15 ± 11.44	0.001
Average	89	38.08 ± 10.98^a^	
Poor	48	42.02 ± 11.24^ab^	
Time since diagnosis			
≤ 12 months	96	36.78 ± 11.52	0.126
> 12 months	82	39.44 ± 11.45	
Medical insurance			
Urban basic health insurance	121	37.83 ± 11.22	0.556
New rural cooperatives medical service	55	38.93 ± 12.04	
Cancer Type			
Lung cancer	53	35.98 ± 10.08	0.130
Gynecologic malignancies	45	40.84 ± 11.95	
Breast cancer	22	41.73 ± 11.41	
Head and neck cancer	21	37.62 ± 12.56	
Gastrointestinal cancer	21	35.43 ± 10.36	
Lymphomas	16	35.50 ± 13.67	

Fisher’s LSD: ^a^*P* < .05 vs. good, ^b^*P* = .05 vs. average

### Development of the preliminary version of CSPDS

According to the semi-structured interviews, self-perceived discrimination could be conceptualized as three subscales: social withdrawal, stigma and self-deprecation. There were 21 items in the original scale. Among them, 9 items were about social withdrawal factor, 8 items about stigma factor, and 4 items about self-deprecation factor; 16 were forward-scored and 5 items were reversed-scored. We revised the wording of 8 items after the first expert consultation. All I-CVIs of the 21 items were >  0.80 after the second consultation. No word amendment was required as the 2 times verbal probing finished (Table 2).

**Table 2:** Item analysis and final version of CSPDS

Draft version of CSPDS	Factor	Item analysis	Final version of CSPDS (Renumber)
1. I will be very happy if someone would like to visit me in the hospital.	F1	√	Item 1
2. Cancer means fewer and fewer friends	F1	√	Item 2
3. Sometimes, I am ashamed when I face other people.	F1	√	Item 3
4. People look down upon cancer patients.	F1	√	Item 4
5. I hate others talking about my disease behind me.	F1	√	Item 5
6. I’d like to talk about my disease with others.	F1	×	
7. I hate someone staring at me.	F1	√	Item 6
8. Having cancer is because of sin.	F2	√	Item 7
9. It would be bad for me if too many people know the state of my disease.	F1	√	Item 8
10. People would not like to approach patients because they believe cancer is a contagious disease.	F2	√	Item 9
11. People would deliberately avoid meeting cancer patients because they think cancer patients will borrow money from them.	F2	√	Item 10
12. People believe that it will bring bad luck to contact cancer patients.	F2	√	Item 11
13. I would not like to go to public place.	F1	×	
14. Many people believe that cancer treatments are effective.	F3	×	
15. Sometimes, I feel I am unlucky.	F3	√	Item 12
16. Sometimes, I feel I am useless.	F3	√	Item 13
17. All family member have provided sufficient care for me since I was ill.	F2	×	
18. I am a burden of my family.	F3	√	Item 14
19. Some people give counterfeit drugs to cheat cancer patients.	F2	×	
20. Cancer patients are vulnerable to be treated unfairly by insurance.	F2	×	
21. Community or village committee have provided sufficient care for me since my disease.	F2	×	

*F1* social withdrawal, *F2* stigma, *F3* self-deprecation

### Item analysis

1. ***Correlational analysis*—**The correlation coefficients of item 6, item 13, item 14, item 17, item 19, item 20 and item 21 were 0.201, 0.267, 0.238, 0.256, 0.194, 0.289, and 0.127, respectively. The correlation coefficients of the remaining 14 items ranged from 0.388 to 0.640 (*P* < .001).
2. ***Cronbach****’****s alpha***—The Cronbach’s alpha of the preliminary scale was 0.781, and it was significantly increased with the removal of item 6, 13, 14, 17, 19, 20 and 21 (See 3.5). The remaining items were renumbered to form the final 14-items CSPDS (Table 2).

### Construct validity and content validity

**Fig. 1:**
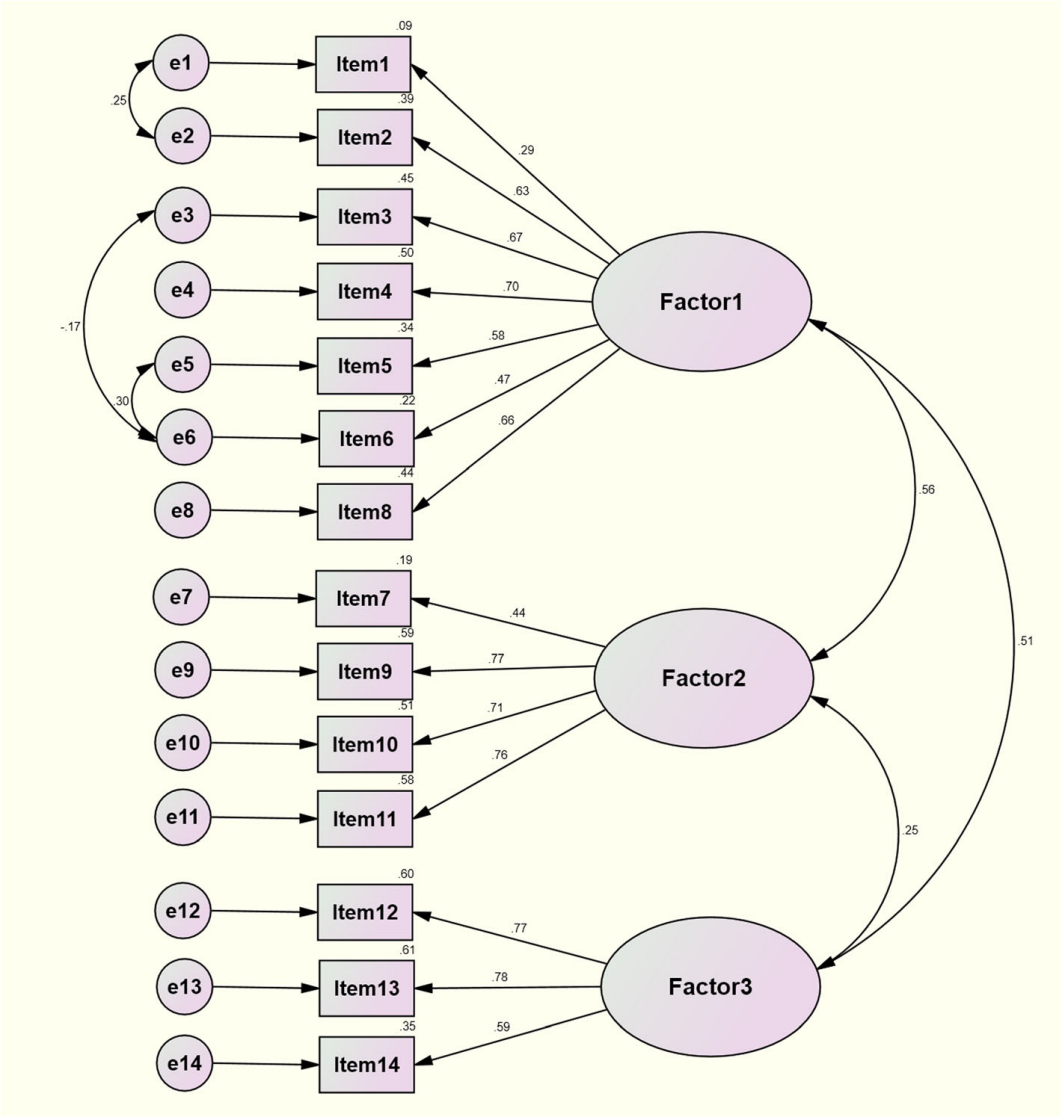
The structural equation modeling of CSPDS

The goodness of fitting of the three-factor model coincided with the initial conception of the scale’s general structure excellently (Fig. 1, Table 3). The fitting degree of the correlated three-factor model was better than the uncorrelated three-factor model. Factor 1 meant “social withdrawal”; it mainly described how cancer patients began to withdraw contact with people after being diagnosed. Factor 2 was labeled “stigma”; it reflected the fact that patients felt their reputation had been unfairly maligned as a result of their disease. Factor 3 was labeled “self-deprecation”; it reflected patients’ negative attitudes toward themselves under multiple burdens derived from their health, mentality, society, finances, or fear of death.

**Table 3:** Fit indices of confirmatory factor analysis for CSPDS

Factor structure	*P*	χ^2^/df	GFI	AGFI	CFI	NFI	TLI	RMSEA
Uncorrelated three-factor model	0.001	1.562	0.916	0.880	0.939	0.851	0.925	0.056
Correlated three-factor model	0.104	1.216	0.935	0.903	0.978	0.889	0.971	0.035
Threshold	> 0.05	< 2	> 0.90	> 0.90	> 0.95	> 0.90	> 0.95	< 0.05

Since I-CVIs of every item in the preliminary CSPDS (21 items version) was ≥0.80, the I-CVIs in the final 14 items version were all ≥0.80 definitely. The correlation coefficients of each item were strongly related to the total scale by Pearson correlation analysis (γ ranging from 0.381 to 0.655, *P* < 0.01). The coefficients between each item and the subscale ranged from 0.471 to 0.839 (*P* < 0.01). Therefore, the CSPDS had a good content validity.

### Reliability

The Cronbach’s alpha for the final CSPDS was 0.829, while those for the three subscales were 0.781, 0.759 and 0.754, respectively. In addition to alphas, the Spearman-Brown coefficients of the total scale and the three subscales were 0.827, 0.803, 0.781 and 0.782, respectively, indicating sufficient internal reliability of the CSPDS. One week after the first interview, a total of 22 patients took part in the second test for test-retest reliability. The test-retest reliability coefficients for the CSPDS and subscales were all excellent, and the ICC of the CSPDS and the 3 subscales were 0.944, 0.926, 0.893, and 0.854, respectively (Table 4).

**Table 4:** Reliability of the CSPDS and three subscales

	Cronbach’s α	Split-half reliability (Spearman-Brown coefficient)	Test-retest reliability	
			ICC	95% CI
CSPDS	0.829	0.827	0.944	0.870–0.976
Social withdrawal	0.781	0.803	0.926	0.831–0.969
Stigma	0.759	0.781	0.893	0.761–0.954
Self-deprecation	0.754	0.782	0.854	0.681–0.937

### Acceptability

The completion time of the final 14-item CSPDS was 6.06 ± 1.80 (range: 3 to 9) minutes. The Flesch Reading Ease of final CSPDS in English was 60.1, and the Flesch-Kincaid Grade Level was 7.5.

### Subgroup-analysis

Regarding the CSPDS total scores, significant differences were found in genders and self-rated health status (*P* < 0.05). The total score of the participants reporting poor self-rated health (42.02 ± 11.24) was higher than that of the participants reporting good health (33.15 ± 11.44; *P* < 0.001) and average health (38.08 ± 10.98; *P* = 0.05). The total score of the participants reporting average health was higher than that of those reporting good health (*P* = 0.02). In contrast, no significant differences were detected in any other characteristics (*P* >  0.05; Table 1).

## Discussion

Our research showed that cancer-related self-perceived discrimination has three main subscales: social withdrawal, stigma, and self-deprecation, therefore the 14-item CSPDS was developed using the classical test theory. There was a high fit degree between the three factor structure and the initial conception (χ^2^/df = 1.216, GFI = 0.935, AGFI = 0.903), the I-CVIs and Pearson correlations of content validity were all at a satisfactory level, which confirmed that the CSPDS had a good instrument validity. It also has a good stability measured by Cronbach’s alpha (0.829), Spearman-Brown coefficient (0.827), and ICC (0.944). Its survey completion time and readability scores are at acceptable levels. Thus, it can be used to assess cancer-related discrimination in terms of social withdrawal, stigma, and self-deprecation.

The CSPDS is more comprehensive and more concise compared with CLCSS and SSS. Although the Cronbach’s alpha of the CSPDS may be lower than the 31-item CLCSS (Cronbach’s alpha = 0.96) and the 21-item SSS (Cronbach’s alpha = 0.94) [23, 24], the CSPDS had excellent test-retest reliability and construct validity, and less items. While the social withdrawal/isolation and stigma subscales and items in the CSPDS are similar to the CLCSS and the SSS, we include the self-deprecation subscale. Most patients, when interviewed, expressed that self-deprecation was a major cause of self-perceived discrimination, therefore, its inclusion could be useful.

The CSPDS has a wider scope of application than the CLCSS and the SSS. The SSS focuses on the influence of the facial tissue defect in patients with head and neck cancer [24]. The CLCSS was used primarily in lung cancer patients with a history of smoking, because researchers considered stigma was based on the belief that smoking is the major cause of one’s own cancer [36]. But lung cancer patients in China may not consider smoking a stigma or a cause of discrimination. The prevalence of smoking is very high in China, therefore, not like in U.S. or in Europe, there is no nationwide strict anti-smoking laws. Even through No-smoking signs are eye-catching in public places, smokers will not obey. These two scales are both limited to a single kind of cancer, or a single cause of cancer. However, the CSPDS can be applied to assess all kinds of cancer and could be a more promising tool of primary screening of self-perceived discrimination.

Notably, the CSPDS shows obvious differences in patients with different self-rated health states and genders. The CSPDS scores of participants who reported poor or average self-rated health state were higher than those who reported good state. It may indicate that perceived discrimination of cancer patients is positively correlated with subjective severity of the disease [36]. The result that women had higher scores than men could be linked to feminine psychological characteristics, social status, family status, and even sexual discrimination, however, we believe cancer is a catalyst for the discrimination against them. Women’s family and social status in poor rural areas is far below the national average level in China [37]. Consequently, when women in poor Southwest China suffer from cancer, the discrimination against them would get worse or be newly genarated. Factors such as feudal thoughts or male chauvinism could lead to the beliefs that ill women are useless and a burden to the family, therefore they become victims of cancer-related discrimination.

Previous studies about cancer-related discrimination focused on workplace and insurance. However, in our semi-structured interviews with the 11 patients, no one described obvious workplace discriminatory behaviors in any form, so we did not include items of workplace discrimination in the preliminary version of the CSPDS. When comparing the participants’ characteristics, results of occupational classification showed negative. This may be connected with Chinese health retirement policy, fair employment policy, and older age sample selection.

In this study, we find no obvious basic medical insurance discrimination. In our semi-structured interviews, 3 interviewees complained that they had experienced insurance discrimination following cancer, so we did set the item “Cancer patients are vulnerable to be treated unfairly by insurance” in the preliminary version, but it was deleted after the item analysis. Our study showed 176 participants used basic health insurance (2 didn’t use medical service were excluded). Chinese basic health insurance schemes have achieved full coverage. It means that more than 1.17 billion people have access to basic health insurance schemes. Chinese basic health insurance system is divided into the Urban and Rural Resident Medical Insurance (URRMI) and the Urban Employee Medical Insurance (UEMI). Chinese government is trying to help ordinary people to pay for their medical costs. In general, Chinese people have an easy access to median low-level medical facilities, and an easy access general hospitals or specialized hospitals [38]. Patients with extreme real-life difficulties can enjoy extra medical subsidies from the government. The Chinese government has also established a Medical insurance for major diseases, to reimburse the high medical expenses of major diseases such as malignant tumors for urban and rural residents. These measures have been further easing the phenomenon that some patients are back to poverty because of those major diseases. Some charities and pharmaceutical companies have also launched charitable assistance programs against high-priced cancer drugs, further increasing access to high-priced cancer drugs for patients with financial difficulties.

In clinical treatment, some cancer patients suffer from social discrimination, which can have a negative impact on their health. The CSPDS would be helpful in discovering this problem in a timely manner. We envisage this measure being properly administered in terminal cancer patients and long-term cancer survivors. The stigma, rejection and isolation cancer patients suffering could come from the public’s fear of death [16]. Terminal cancer patients are always in poor physical condition, and confronted with the threat of death. Our results suggest that the patients reporting poor self-rated health suffered more discrimination than those reporting good health or average health. So terminal cancer patients may experience more perceived discrimination. Besides, perceived discrimination is a negative psychological factor. Long-term cancer survivors could perceive more negative impacts of cancer. These perceptions appear to influence, or are potentially influenced by, physical and mental health-related quality of life [39]. Therefore we think that long-term cancer survivors also may experience more perceived discrimination.

Having taken those into consideration, the CSPDS may be more appropriate for preliminary screening of perceived discrimination. Furthermore, the CSPDS may help to develop targeted patient education, psychological diagnosis, psychological counseling or treatment, and social interventions, so as to improve patients’ quality of life and social functioning as well as to prolong survival time among cancer patients.

As an exploratory study, this study has relatively few inpatients from a single center. The samples come mainly from South-western China, where is underdeveloped, so this result represents only patients in Southwest China. Future studies should cover multiple areas and centers with larger samples, comparing the self-perceived discrimination levels in different types of cancer, and evaluating the correlations between self-perceived discrimination and other negative psychological states (e.g. anxiety, depression, disease uncertainty, post-traumatic stress disorder, etc), so as to provide more abundant evidence for cancer-related discrimination. Furthermore, as there is no authoritative or widely agreed standard measure in this field, we did not assess the criterion-related validity of the CSPDS. Moreover, if any researcher need to use this measure, we allow him/her to translate it into other languages and further verify the reliability and validity, or revise it.

Further studies will be required to identify the precise relationship between gender and cancer-related discrimination. In view of possible workplace and insurance discrimination, young cancer patients and long-term survivors should be studied to determine whether workplace discrimination is in existence, and how it would influence the patient. More varieties of commercial insurance discrimination, such as health insurance, life insurance and endowment insurance, will also be considered in later stuies.

## Conclusion

The CSPDS is accurate and reliable in our study, which could be used for assessing self-perceived discrimination among cancer patients and as a basis for patient education, counseling, social interventions, and psychotherapy in the future, at least in Southwest China.

## Abbreviations

AGFI: Adjusted goodness of fit index
CFA: Confirmatory factor analysis
CFI: Comparative fit index
CLCSS: Cataldo Lung Cancer Stigma Scale
CSPDS: Cancer Self-Perceived Discrimination Scale
GFI: Goodness of fit index
HBOC: Hereditary breast and ovarian cancer
ICCs: Intra-class correlation coefficients
I-CVIs: Item-level content validity indexes
LSD-t: Fisher’s least significant difference test
NFI: Normed fit Index
RMSEA: Root mean squared error of approximation
SEM: Structural equation modeling
SSS: Shame and Stigma Scale
TLI: Tucker-Lewis index
χ^2^/df: Chi-Square divided by degrees of freedom
